# Integrative Music Therapy for Depression-related Disorders (iMTDep) – study protocol for a randomised controlled trial

**DOI:** 10.1186/s13063-026-09864-4

**Published:** 2026-06-19

**Authors:** Esa Ala-Ruona, Tiia-Liina Raittila, Marianne Taipale, Nandhini Natarajan, Martin Hartmann, Anastasios Mavrolampados, Alessandro Ansani, Eliisa Löyttyniemi, Jaakko Erkkilä

**Affiliations:** 1 The Centre of Excellence in Music, Mind, Body and Brain, University of Jyväskylä, Jyväskylä, Finland; 2 Department of Biostatistics, University of Turku and Turku University Hospital, Turku, Finland

**Keywords:** Music therapy, Depression, Anxiety, Work-related stress, Randomised controlled trial, Clinical study, Clinical improvisation, Music listening, Vibroacoustic treatment

## Abstract

**Background:**

Depression, anxiety and work-related stress affect a significant number of adults disrupting their productivity, ability to function and attend work. These highly comorbid disorders tend to be difficult to treat effectively, and more information about integrative treatment approaches is needed. This study investigates the effectiveness of music therapy in treating depression-related disorders.

**Methods/design:**

The intervention is targeted at people of 18–65 years of age who are in employment, in their studies, temporarily unemployed, on short-term sick leave or rehabilitation allowance and suffer from depression-related disorders, which include one or several of the following: depression, anxiety, work-related stress or exhaustion. The interventions applied are Integrative Improvisational Music Therapy (IIMT) with or without the additional elements of music listening and vibroacoustic treatment. All sessions will begin with a preparative exercise called Resonance Frequency Breathing to enhance the effect of the therapy. All participants will receive the intervention for 6 weeks (60-min therapy session twice a week). The participants will be randomised into four groups that will have different modifications of the intervention, and the group without additional elements will serve as a waiting-list control group. The primary outcome will measure psychological distress. Secondary outcomes will address depression, anxiety, exhaustion, burnout, health-related quality of life and challenges in recognising emotions (alexithymia).

**Discussion:**

The trial will elevate our understanding of the efficacy of integrative improvisational music therapy for depression-related disorders. Results will give more information about the combinations of additional elements used as part of the intervention. Extensive dataset will allow us to investigate the dynamics of therapeutic change and the factors that predict certain responsiveness to the intervention or explain the effects.

**Trial registration:**

Prospectively registered on ISRCTN on 12.04.2024 (ISRCTN26812986). Study was approved by the Regional Medical Research Ethics Committee of Wellbeing Services County of Central Finland 15.02.2024 (ref: 7U/2023).

**Supplementary Information:**

The online version contains supplementary material available at https://doi.org/10.1186/s13063-026-09864-4.

## Administrative information

**Table:** 

Title {1}	Integrative Music Therapy for Depression-related Disorders (iMTDep) – Study Protocol for a Randomised Controlled Trial
Trial registration {2a and 2b}	ISRCTN26812986 https://doi.org/10.1186/ISRCTN26812986
Protocol version {3}	The current protocol is version 2 (date of approval: 20/04/2026). Recruitment started 05.06.2024 and is estimated to be complete by the end of December 2026
Funding {4}	This study is funded by the Research Council of Finland; Centre of Excellence in Music, Mind, Body and Brain (Grant number 346210)
Author details {5a}	Esa Ala-Ruona^1^, Tiia-Liina Raittila^1^, Marianne Taipale^1^, Nandhini Natarajan^1^, Martin Hartmann^1^, Anastasios Mavrolampados^1^, Alessandro Ansani^1^, Eliisa Löyttyniemi^2^ & Jaakko Erkkilä^1^ ^1^The Centre of Excellence of Music, Mind, Body and Brain, University of Jyväskylä, Jyväskylä, Finland ^2^Department of Biostatistics, University of Turku and Turku University Hospital, Turku, Finland
Name and contact information for the trial sponsor {5b}	University of Jyväskylä (FI02458947) Prof. Petri Toiviainen, Centre of Excellence in Music, Mind, Body and Brain, Department of Music, Arts and Culture Studies, P.O. BOX 35, FI-40014 Jyväskylä, Finland
Role of sponsor {5c}	The funder and the sponsor had no role in the design of this study, and will not have any role during its execution, analyses, interpretation of the data, or decision to submit the results
Composition of the coordinating centre and trial steering committee {5d}	The study is conducted and coordinated by the iMTDep research group that runs the trial and provides day to day support for the interventionists. This group is in weekly contact with the larger MMBB research group, which provides support and oversight. The trial steering committee meets once a year to discuss the progress of the study. The steering committee consists of three experts, who are from the fields of psychiatry, psychotherapy and neurology. There are also technical staff to provide support in technical issues. The IT services and the data administration and protection services and infrastructure of the University of Jyväskylä are also there to support the team when needed

## Introduction

### Background and rationale {6a}

Depressive, anxiety and exhaustion disorders affect a significant proportion of adults [1, 2]. These disorders disrupt an individual’s ability to function normally, lead to reduced productivity, heightened work absenteeism, and more frequent medical consultations. Although various treatment options are available, the comorbid nature and varied presentation of these disorders make them difficult to treat effectively. Thus, an integrative approach that is tailored to the individual is essential. In this study, we propose Integrative Improvisational Music Therapy (IIMT) [3] as one such method and will study its efficacy in different variations.

According to the Finnish Institute for Health and Welfare [4] depressive disorders are the most common mental health conditions and a major public health issue affecting working age individuals in Finland. They are characterized by feelings of sadness and hopelessness and a loss of pleasure or interest in activities. Physical symptoms like loss of appetite and excessive sleep are also associated with them [5]. Their prevalence among Finnish adults is estimated at 5–7%, and both their personal and societal burden are significant [6]. They are the single largest cause for disability pension in Finland [4].

Anxiety disorders are another common mental health condition in Finland. They are characterised by excessive fear and worry not only about specific but also everyday situations. Physical symptoms can include sweating, trembling, shaking; experiencing abdominal pain or nausea; and having difficulties concentrating and performing other cognitive tasks [7]. The prevalence of anxiety disorders is more difficult to estimate, as they are highly comorbid with other mental disorders and often remain undiagnosed. However, population-based surveys suggest that the lifetime prevalence of anxiety disorders among adults aged 18 to 64 is 6.6% [8]. Anxiety and depressive disorders are often comorbid. A survey by Kessler et al. [9] reported that 47.5% of individuals who had experienced major depressive disorder at some point in their lives also had a lifetime history of one or more anxiety disorders. The comorbidity of depression and anxiety is due to the overlap of symptoms and the shared structural and functional pathways of changes in the brain that are associated with these disorders [10].

Another condition called exhaustion disorder may develop as a result of prolonged stress [11]. It is characterised by physical and emotional fatigue often resulting from work-related stress. While depression and anxiety are classified as disorders by the Diagnostic and Statistical Manual of Mental Disorders (DSM), exhaustion is not. Thus, according to Finnish Current Care Guidelines, exhaustion is currently not recognised as a separate disorder in Finland, which complicates the process of seeking and receiving treatment. However, research conducted in Sweden found that fatigue was inadequately addressed by adjustment disorder, an existing stress-related diagnosis. As a result, they proposed provisional criteria for a new condition called ‘exhaustion disorder’. Conceptually akin to the construct of burnout, exhaustion disorder was incorporated into the Swedish version of the International Classification of Diseases (ICD-10) in 2005, classified under F43.8A Reaction to severe stress. Since its introduction, the diagnosis of exhaustion disorder is almost as prevalent as that of major depression in Swedish healthcare settings [2].

The terms ‘exhaustion disorder’ and ‘burnout’ are often used interchangeably and in this study, we will treat them as the same condition. In a report by de Hert [12], the prevalence of burnout in the European Union countries ranges from 4,3% in Finland to 20,6% in Slovenia. The study also indicated that an increase in workload correlates positively with burnout. According to Finnish Institute of Occupational Health [13] approximately one in four Finns have an increased risk for occupational burnout and severe burnout concerned 10% of people in 2024. These people experience symptoms of burnout constantly but may still be at work. While exhaustion disorder has distinct symptoms, individuals affected by it have reported higher levels of anxiety and depression, often meeting the criteria for both conditions simultaneously [2, 14].

Thus, depressive symptoms are common in all these conditions: in anxiety, due to its comorbid relationship with depression [15] and in exhaustion, as many of the symptoms of exhaustion disorder overlap with that of major depressive disorder [16]. The relationship between depression, anxiety, exhaustion exist not only through aetiology and pathophysiology but also because treatment practices used for classified disorders (depression, anxiety) are used for treating non-classified disorders (exhaustion) as well.

While pharmacological treatments can be effective in treating conditions with a clear diagnosis, they are not as effective in treating conditions with complex comorbidities. Additionally, the use of antidepressants (the most commonly prescribed pharmacological treatment for depression) is associated with a variety of side effects which are often underreported in clinical trials [17]. Hence, more integrated treatment approaches incorporating psychosocial support also need to be adopted that not only alleviate the symptoms of the disorder but also do not cause any harm.

In Finland, depression is usually treated with psychotherapy that has proven to be effective, with medication or with a combination of the two [6]. According to the Current Care Guidelines, these treatments are equally effective for mild and moderate depression, and their simultaneous use often yields the best outcomes. As much as pharmacological treatments provide symptomatic relief, psychotherapy helps clients understand the root causes of their symptoms, process their emotions and provide coping strategies which enhance long term wellbeing.

Music therapy is one form of psychotherapy that can be used to treat and manage depression and other mental health conditions [18]. It uses music to facilitate non-verbal expression in clients who may struggle to express themselves verbally. It is relaxing, creatively engaging, and is associated with less social stigma [19, 20]. There are two Cochrane reviews on the effect of music therapy for depression [21, 22] with promising findings.

At the University of Jyväskylä (JYU), we have conducted two RCTs on the effect of music therapy on depression in adults [23, 24]. Both were based on the clinical model of Integrative Improvisational Music Therapy (IIMT) which involves active music making through musical improvisation to facilitate self-expression in clients. In IIMT, two musical instruments – a djembe drum and a piano – are used, with both the client and therapist each having their own. All musical expression and interaction take place using these instruments, and is based on clinical improvisation, meaning the client does not need any previous musical experience or specific skills. In addition to clinical improvisation, verbal dialogue plays an important role in the process. Conversations can introduce personal themes and experiences that are then further explored musically, while improvisation can elicit emotions, memories, and associations that are further processed through verbal reflection. This turn-taking between improvisation and verbal processing forms a dialogue in which both modes of expression enrich one another. In this way, the therapeutic process becomes more dynamic and productive. The principles and theory of the IIMT have been described by Erkkilä et al. [3].

In this study, we aim to evaluate the effectiveness of music therapy in alleviating symptoms of depression, anxiety, and exhaustion among individuals of working age. Both previously conducted RCTs showed significant improvement on several outcomes measured, including scores of depression and anxiety. The clinical model of IIMT will be used in the current study as well. Specifically, we will assess the impact of two additional elements to the current IIMT model: music listening (ML) and vibroacoustic treatment (VAT). Participants with symptoms related to depression, anxiety, and/or exhaustion disorder will be recruited and randomly allocated to one of four conditions: (A) a waiting-list control group, which will receive standard IIMT after the waiting-list period; (B) ML + IIMT; (C) VAT + IIMT; and (D) VAT + ML + IIMT. Measures of psychological distress, depression, anxiety, and exhaustion will be collected before and after the intervention, as well as in follow-ups at 6-month and 12-month timepoints.

## Objectives {7}

The aim of the study is to focus on depression and anxiety symptoms of individuals of working age and to expand the scope to cover exhaustion as well. This RCT’s intervention is targeted at adult individuals who are in employment, in their studies, temporarily unemployed, on short-term sick leave or rehabilitation allowance and have one or various of the following disorders or conditions: depression, anxiety, work-related stress or exhaustion.

Objective 1: To examine whether IIMT, offered in four different modifications, improves the participants’ condition relative to the control/TAU (Treatment as usual) group. We also aim to find out whether any of the modifications of the IIMT are more effective than plain IIMT.

Objective 2: To understand whether certain musical responsiveness or personality profile explains a client’s improvement in music therapy.

Objective 3: To increase the understanding of the dynamics of therapeutic change by IIMT in its original form and with selected modifications.

Objective 4: To increase the understanding of the essence, mechanisms and trajectories of work-related stress and burnout and their relationship to depression and anxiety.

Hypothesis 1: All the experiment groups (ML + IIMT, VAT + IIMT, VAT + ML + IIMT) improve significantly relative to the control/TAU group.

Hypothesis 2: ML + IIMT group improves significantly better than the IIMT only group.

Hypothesis 3: VAT + IIMT group improves significantly better than the IIMT only group.

Hypothesis 4: VAT + ML + IIMT group improves significantly better than ML + IIMT, and VAT + IIMT groups.

## Trial design {8}

The study is a randomised 2 × 2 factorial trial, using four conditions: IIMT, IIMT with an additional component of music listening (ML), IIMT with an additional component of vibroacoustic treatment (VAT), and IIMT with the additional elements of both ML and VAT. A superiority design is adopted with the aim of testing whether the intervention, in different conditions, demonstrates statistically and clinically superior outcomes compared with the comparator.

All sessions regardless of the condition will begin with a preparing exercise called Resonance Frequency Breathing (RFB) [24]. There will be a waiting-list control group to whom the plain IIMT will be offered after six weeks of baseline measurements, allowing a within-subject comparison between baseline and treatment conditions. Each group is offered a therapy process consisting of 12 sessions, conducted twice a week for 60 min each.

**Fig. 1:**
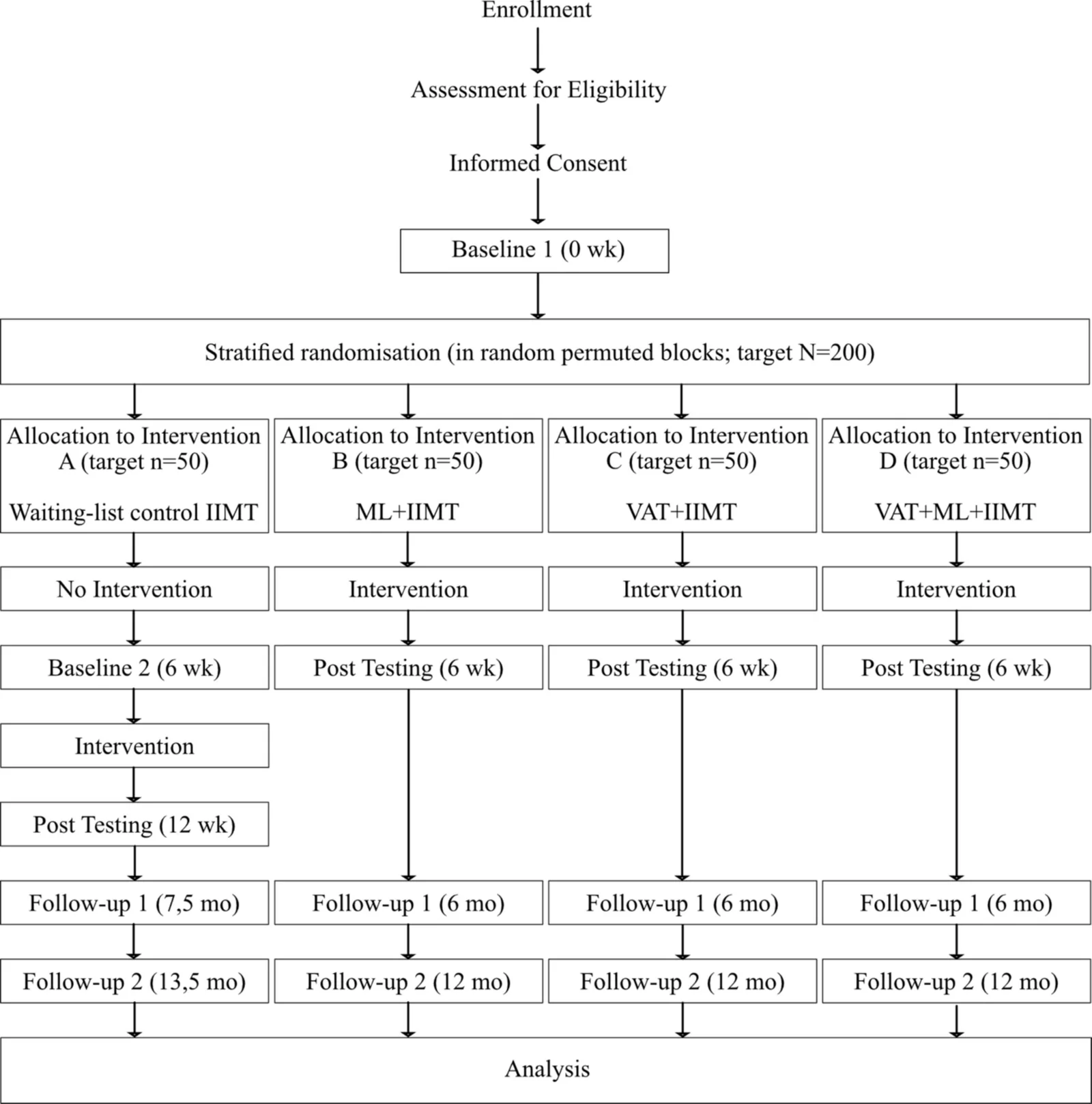
The flow-chart of the iMTDep RCT

Outcomes will be measured before and after the start of the therapy and at two follow up points, 6 months and 12 months after the start of the therapy. For Condition A there will be additional baseline 2 measurements before the start of the therapy (see Fig. 1).

## Methods: Participants, interventions and outcomes

### Study setting {9}

The study will be conducted in an academic setting at the JYU Music therapy clinic for research and training at the University of Jyväskylä, Finland. All the data will be collected in Finland.

### Eligibility criteria {10}

Participants are included if they score 15 or higher on MADRS [25] and/or 7 or higher on HADS-A [26] and/or 19 or higher on KEDS [11]. Exclusion criteria are psychosis, a recognized personality disorder, acute substance abuse (measured with AUDIT [27]), difficulty in engaging in the study and too severe depression (e.g. self-destructive behaviour or chronic depression) for participation in therapy. In addition, the participants are excluded if they are pregnant or have a pacemaker, as those conditions are contraindications for participating in the VAT.

The Research Centre has former experience of conducting RCT studies [23, 28]. The facilities and equipment have been technically renewed in 2021–2023 to meet the current research needs of The Centre of Excellence in Music, Mind, Body and Brain (MMBB). All assessments will be performed by qualified health care personnel with experience of assessment, evaluation and psychometric testing. All interventions will be performed by qualified music therapists that have undergone uniform protocol training for working as interventionists in this study setting. Adherence to the intervention protocol will be monitored in clinical supervision.

### Who will take informed consent? {26a}

The assessors will collect the informed consent from participants, after they are screened and assessed for eligibility. If they meet the inclusion criteria (sufficient score in the screening with MADRS, HADS-A and/or KEDS) and do not meet any of the exclusion criteria, they will be asked to give their consent to participate in the study. After giving their consent, the other baseline measurements are conducted in the same session.

### Additional consent provisions for collection and use of participant data and biological specimens {26b}

Not applicable; no additional consent is needed, and no biological samples are collected.

## Interventions

### Explanation for the choice of comparators {6b}

The Condition A serves as a waiting-list control group, and they receive their intervention after a 6-week waiting period. This group will serve as the comparator for the primary intervention. Throughout the study all participants in all groups will receive regular healthcare (treatment as usual).

### Intervention description {11a}

The intervention consists of 12 therapy sessions (60 min twice a week) in an individual setting. The intervention is based on a treatment model developed at our unit and previously utilised in two RCTs [23, 28]. This intervention is called Integrative Improvisational Music Therapy (IIMT). In this trial, there are four different conditions, which are modifications of IIMT (except for Condition A, which consists solely of IIMT). Despite these modifications, IIMT remains the primary intervention. The modifications in Conditions B, C, and D occur at the beginning of the sessions and are as follows: ML + IIMT (Condition B), VAT + IIMT (Condition C), and VAT with ML + IIMT (Condition D). Detailed descriptions of all conditions are provided in the following section.

#### IIMT only

Condition A (IIMT only) begins with a 7-min RFB exercise [24]. Following this, the session proceeds with IIMT activities, which include clinical improvisation and discussion. The total session duration is 60 min, with approximately 40 min dedicated to IIMT activities. In the IIMT model, the length and number of improvisations, as well as the amount of discussion, are not predefined but are determined intuitively by the therapist and client. The musical instruments used in IIMT are identical for both participants: a djembe drum and a digital keyboard with piano sounds for both the therapist and the client, ensuring equal instruments for both improvisers.

#### IIMT with music listening

Condition B begins with a 7-min RFB exercise and includes an additional component of music listening prior to IIMT. The recommended duration for this component is 15 min but may vary slightly depending on the situation. Music selection can be made by the client or the therapist, depending on the current needs of the moment. A typical starting point is to listen to music, which is somehow meaningful or currently important to the client.

#### IIMT with vibroacoustic treatment

Condition C begins with a 7-min RFB exercise and includes an additional component of vibroacoustic treatment used prior to IIMT. The recommended duration for this component is 15 min, though it may vary slightly depending on the dynamics of the moment.

#### IIMT with vibroacoustic treatment combined with music listening

Condition D begins with a 7-min RFB exercise and combines the additional elements of vibroacoustic treatment and music listening prior to IIMT. The recommended duration for this component is 15 min, though it may vary slightly depending on the dynamics of the moment.

### Integrative improvisational music therapy (IIMT)

IIMT is a music therapy model [3], which consists of clinical improvisation usually with two similar pianos and djembe drums in a client-therapist dyad, and reflection of the experience in verbal discussion. It originally roots back to psychoanalytical ideas about the meanings of music in improvisational music therapy which can be used in a psychodynamic context as well as in psychodynamic psychotherapy. Improvisational music therapy, such as IIMT, is suitable for all client groups, and does not require specific musical training from the client. The model can be successfully applied even to clients with limited ability for verbal expression. As a form of non-verbal expression and interaction, clinical improvisations can lead to the emergence of the client’s abstract and unshaped mental contents from the subconscious to the conscious level. These experiences are often accompanied by emotional aspects. Adults with typically developed cognitive skills and capacity of abstract thinking can connect symbolic and experience-based mental contents to their musical expression in the context of clinical improvisation [29]. The effectiveness of IIMT has been proven to be significant compared to standard care with depressed patients, and to be enhanced by adding a breathing exercise called Resonance Frequency Breathing (RFB) [28, 30]. In this study, RFB is integrated in the beginning of every therapy session to prepare the client for improvisation and enhance the effect of IIMT. The individual optimal breathing pace is defined before the start of the therapy process by conducting an individual breathing test for each participant. The test is based on heart rate variability (HRV) measurement [24].

### Music listening

Receptive techniques [31] are one of the main intervention families in music therapy consisting of various models and methods based on music listening. In our study, we will utilise both sedative and stimulating musical stimuli according to a client’s current needs. There are a few factors, which determine sedative and stimulating musical experience. According to the most universal principles we will put attention to certain basic determinants such as high frequencies (tension related), and low frequencies (relaxation related), strong rhythm (energy and activity) versus rhythmically neutral music (calmness), and loud music (arousal and awareness) versus soft music (pacifying, calming, relaxing) [32]. According to research evidence [32] most effective music to use is the music familiar and preferred by the client. However, sometimes also the therapist must make a choice – for instance, when a client asks for it. In this study, both music selection types are allowed. Both instrumental and vocal music are possible. In music listening intervention, therapeutic relationship (dialogue) between client and music therapist is involved, which makes the intervention music therapeutic according to the definition by Bruscia [33]. In addition to goals such as relaxation and stimulation, music listening is widely used in music therapy for evoking images and emotional memories, which often are connected to therapeutically meaningful themes [31, 34].

### Vibroacoustic treatment with or without music listening

Vibroacoustic treatment can be considered as a form of receptive music therapy, which can be applied with or without music listening. It uses low frequency (20–120 Hz) sinusoidal sound vibration, delivered through a device with loudspeakers or transducers. Vibroacoustic treatment can be directed locally or as a full-body stimulation. The main concept of vibroacoustic treatment is sympathetic resonance, which occurs in the body when the sound vibration affects the certain resonant body parts [35]. The vibrating effect in a body may support either relaxation or activation, depending on the speed of pulsation (amplitude change over time). Also, listening to music can be calming down or activating, for instance, evoking imagery or triggering some memories. In vibroacoustic treatment both psychological and physiological processes can be observed in the same situation. This allows reflective therapeutic discussion on the levels of thoughts and memories (cognitive), images (symbolic), feelings (emotional), and bodily sensations (physiological). An integrative view to a client’s experiences offers a possibility to work on different thematic content, and to further process these in active music therapy, in this case, by using IIMT [32, 35, 36]. In this study, vibroacoustic treatment is offered for 15 min as 40 Hz sinusoidal sound vibration with 6,8 s curve of amplitude change. The only adjustable parameter is the overall volume level of the stimulation, which will be individually set to a comfortable level.

#### Criteria for discontinuing or modifying allocated interventions {11b}

The participants have the right to withdraw from the study at any point. The need to discontinue the intervention may arise, for example, due to the participant’s strongly weakened condition, physical limitations or changes in life situation. The possibility of modifying or discontinuing the intervention will be discussed between the assessor, supervisor, therapist and participant, considering participants’ wishes and any safety-related concerns. Therapists participate in group supervision, where possible modifications of the intervention or discontinuation of the intervention can be further discussed. The consulting psychiatrist may be contacted if needed.

#### Strategies to improve adherence to interventions {11c}

All the clinically qualified music therapists are obligated to complete an additional intervention training consisting of theory, practical demonstrations, workshops, collaborative reflection, technical guidance, and independent rehearsals in dyads or triads. The training period lasts approximately 5 months and is conducted on weekly basis, four hours per time. After the training the therapists participate in weekly group supervision sessions (for four hours each) throughout their clinical work. The adherence to treatment protocol is assessed by monitoring the video recordings of the therapy sessions frequently as part of the supervision. The therapists fill out a session questionnaire after each therapy session for self-reflection and reporting of their own observations. Individual clinical supervision may be arranged in special occasions as needed for ensuring the adherence to protocol and treatment fidelity.

#### Relevant concomitant care permitted or prohibited during the trial {11d}

During the trial, the participants are permitted to receive all standard treatment, and their other ongoing therapies are not prohibited. Possible changes in care, rehabilitation and/or complementary treatments will be recorded by the assessors during the appointments.

#### Provisions for post-trial care {30}

This study itself is not responsible for offering post-trial care for the participants, but when needed, the participant is informed about the local psychiatric and health care services they can contact to receive additional care. The consulting psychiatrist can also be contacted to evaluate a participant’s situation.

#### Outcomes {12}

All the primary and secondary outcome measures will be assessed at baseline, post and follow-up testing timepoints (see Table 1 below).

**Table 1:**
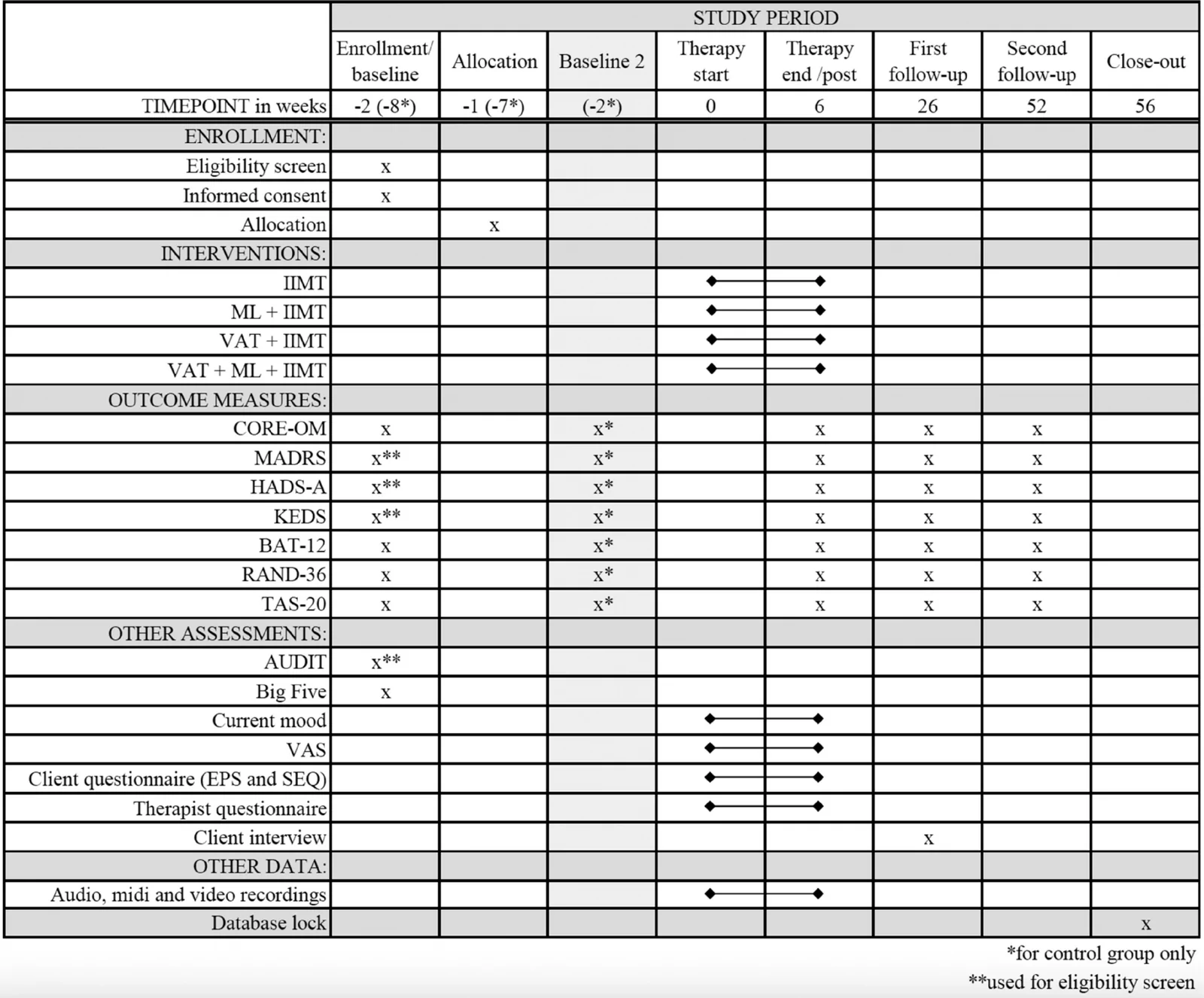
Outcomes according to study period

### Primary outcome

The primary outcome measure of this study will be Clinical Outcomes in Routine Evaluation – Outcome Measure (CORE-OM) [37]. It is a 34-item self-report measure that will be used to address the distress and life/social functioning of the participants. The Finnish version of CORE-OM has been found to be reliable and valid assessment tool for psychological distress in both clinical and non-clinical settings [38].

### Secondary outcomes

There will also be several secondary outcome measures. The Montgomery Åsberg Depression Rating Scale (MADRS) (also used for screening for eligibility) [25] will be used to assess the severity of depressive symptoms. A 10-item questionnaire is based on an interview conducted by a clinician. The MADRS has high joint-reliability, it has been shown to be sensitive to change, and it has also been demonstrated to have predictive validity for major depressive disorder [39]. The anxiety subscale (HADS-A) (also used for screening for eligibility) of the Hospital Anxiety Depression Scale (HADS) [26] will be used to assess the level of anxiety. The HADS is a well-established self-report questionnaire that has been widely used for assessing anxiety and depression in a non-psychiatric adult population. The HADS-A consists of seven items. The Finnish translation has been shown to be reliable and internally consistent short and user-friendly instrument [40].

Work-related stress and exhaustion will be assessed with the Karolinska Exhaustion Disorder Scale (KEDS) (also used for screening for eligibility) [11] and the Burnout Assessment Tool (BAT) [41]. The KEDS is a nine-item summated self-rating scale that has been developed to assess symptoms of stress-included exhaustion disorder. The scale has shown to be unidimensional and internally consistent [11]. The BAT is a self-report questionnaire developed to measure burnout [41]. The original BAT consists of 23 items. The shortened 12-item version BAT-12 has shown to have similar psychometric properties than the full version and is well suited for research purposes [42]. There are yet two versions available of BAT-12, a work-related and a general version, that will both be utilised in this study because the work-related version cannot be filled by those currently not working.

RAND-36 will be used to assess the health-related quality of life. The focus of the 36-item survey is on eight quality of life -related domains that are physical functioning, bodily pain, role limitations due to physical health problems, role limitations due to personal or emotional problems, emotional well-being, social functioning, vitality, and general health perceptions. The Finnish version of the RAND-36 has also found to be reliable, valid and adequate in measuring the health-related quality of life [43]. Toronto Alexithymia Scale (TAS-20) will be used to assess the possible alexithymia since it is a reliable and valid measure of alexithymia [44]. The Finnish version of TAS-20 has been shown to operate satisfactorily [45].

### Other assessments

The measures conducted during the recruitment of the participants will also be the ones that determine participants’ eligibility for the study. In addition to the outcome measures demographics such as gender, age and socio-economic background will be inquired at the baseline testing. The health care professional responsible for testing will collect the health background information, such as medication and ongoing or planned treatment and therapies. Possible changes in treatment and care during the research process will also be recorded in testing appointments. The 50-item version of Big Five Inventory will be used to assess the personality dimensions of the participants. The Big Five or the five-factor model is a working consensus regarding the descriptive structure of personality traits [46]. Acute substance abuse will be screened by the Alcohol Use Disorders Identification Test (AUDIT) (also used for screening for eligibility) that has been found to be valid and reliable in identification of harmful and hazardous alcohol consumption [27]. The Big Five and the AUDIT inventories will be used in the baseline assessment only.

In addition, participants receiving vibroacoustic treatment will complete Visual Analogue Scale (VAS) before and after VAT to assess their condition at that particular moment. All the clients and therapists will also answer online questionnaires at the end of each session to evaluate the session and assess their emotional processing. The client questionnaire includes Emotional Processing Scale (EPS) and Session Evaluation Questionnaire (SEQ). The therapist questionnaire includes SEQ and additional open questions about each session. A subset of data about participants’ experiences will be collected through interviews to deepen the understanding and to interpret the results of the outcome measures. All the participants will evaluate their current mood on scale of 1–10 in the beginning of every therapy session.

#### Participant timeline {13}

There are overall four to five testing timepoints in this study: during the recruitment of the participants (baseline), 6 weeks after the beginning of the intervention (post-intervention), 6 months after the beginning of the intervention (first follow-up) and 12 months after the beginning of the intervention (second follow-up). The waiting-list control group will have an additional testing timepoint (baseline 2) at 6 weeks after the initial baseline tests before starting the therapy. This will also act as a new baseline for the intervention.

#### Sample size {14}

We will recruit 200 working-age adults (18–65 years of age) suffering from one or several of the following disorders: depression (ICD-10: F32 or F33), anxiety (ICD-10: F41), or exhaustion. The sample of participants will be randomised into four groups (50 + 50 + 50 + 50) of which two groups (*n* = 100) will receive vibroacoustic treatment (VAT) and two groups (*n* = 100) not whereas two groups (*n* = 100) will receive music listening (ML) and two groups (*n* = 100) not (see Table 2).

**Table 2:** Factorial design

	VAT yes	VAT no	
ML yes	50	50	*n* = 100
ML no	50	50	*n* = 100
	*n* = 100	*n* = 100	

For each condition, the selected sample size provided a statistical power of 0.985 for detecting a medium standardised effect size of Cohen’s d = 0.5 in a mixed-model analysis [47], with a 2-tailed significance level of *p* < 0.05 and intra-participant correlation of ρ = 0.6. For the comparison between IIMT only vs waiting-list control, the selected sample size provided statistical power of 0.9 for detecting a medium effect size (d = 0.23) in a repeated measures t-test, with a 2-tailed significance level of *p* < 0.05. These high-power levels ensure a robust detection of clinically meaningful effects, even after accounting for potential participant dropouts, while reducing the likelihood of Type II errors. Also, the selected sample sizes also retain adequate power (> 0.8) to detect smaller effects (e.g., d = 0.2), thus providing sensitivity to subtle group differences.

#### Recruitment {15}

We will recruit 200 adults of working age who are 18–65 years of age, assessed with one or more of the following conditions: depression (ICD-10: F32 or F33), anxiety (ICD-10: F41), or exhaustion. Participants will be recruited from the middle-Finland area through newspaper announcements, media bulletin, flyers in psychiatric policlinics, health centres and public institutions (based on permissions), and social media. We will also inform local health care professionals about the study to enable recruitment through word of mouth.

The advertisements include the named contact people for the study. Those individuals interested in participating in the study may contact the named people by phone or email and enrol in the study if they wish. They are then informed about the study in more detail. Initially, the participants receive detailed information about the study, a research bulletin and a consent form, to make sure they have sufficient time to familiarise themselves with it before coming to the first appointment and making their final decision of participation. Screening will be conducted in the first assessment appointment as described earlier in this document. The participants have the opportunity to contact the research group at any time during the process and ask for additional information. The local health care professionals will be informed about the study. A separate permission for contacting the participant for further studies will be asked on the consent form.

## Assignment of interventions: allocation

### Sequence generation {16a}

The stratified randomisation will be performed using the random permuted block method.

The stratification factors will be age group, gender, employment status and health condition. An external biostatistician will create the program for randomisation. The randomisation table will be created using the SAS software, Version 9.4 of the SAS System for Windows (SAS Institute Inc., Cary, NC, USA). The table will be used to set up the randomisation module on REDCap, and REDCap will be used further for the randomisation. An external biostatistician does not have any contact to the study participants, and the sequence is kept concealed from those who do. Study data will be collected and managed using REDCap electronic data capture tools hosted at the University of Jyväskylä [48, 49]. REDCap (Research Electronic Data Capture) is a secure, web-based software platform designed to support data capture for research studies, providing 1) an intuitive interface for validated data capture; 2) audit trails for tracking data manipulation and export procedures; 3) automated export procedures for seamless data downloads to common statistical packages; and 4) procedures for data integration and interoperability with external sources.

### Concealment mechanism {16b}

Concealment is implemented by using central allocation through REDCap.

### Implementation {16c}

The participants will be enrolled by the assessors using the aforementioned screening tests. The randomisation will be carried out by the PI who will then inform another staff member about the participant’s allocation. The staff member will inform the next available therapist about the participant’s allocation, and the therapist contacts the participant. The participants are not informed about the presence of other conditions. For the waiting-list condition, the participants are invited for another screening (baseline 2) six weeks post the initial screening.

## Assignment of interventions: blinding

### Who will be blinded {17a}

The assessors will be blinded about the participants’ randomisation to different conditions throughout the assessment points.

### Procedure for unblinding if needed {17b}

Only the assessors are blinded and since they are external members not involved in the study in any other way, there will be no need for unblinding. In case of the participant revealing their allocation by chance, it will be recorded. However, the assessment will proceed as planned.

## Data collection and management

### Plans for assessment and collection of outcomes {18a}

The assessment is conducted in four different testing timepoints (except Condition A that has an extra testing timepoint before the intervention). All outcome measures are completed in all timepoints. The outcome data will be collected by the assessors using paper questionnaires. The assessors are healthcare professionals that have been trained to conduct the study protocol and procedures. The data will be stored in REDCap by researchers. REDCap software is suitable for collecting sensitive data. The software requires individual multi-factor authentication and only authorized staff that have a specific reason to handle the data will have access to it. The access to and collecting and storing of the data as well as access control will be monitored together with the University of Jyväskylä (JYU) IT services and information administration. In REDCap, the PI can assign different access rights to different research group members. The final complete data will be stored in a server that is subject to the JYU information security policy.

### Plans to promote participant retention and complete follow-up {18b}

The participants’ eligibility and ability to commit to the study is assessed at screening to ensure the fit to the protocol. The intervention is designed to meet the needs of the target group thus promoting participant retention and completing the intervention and the whole study. Participants are also encouraged to complete the study. The study will be implemented with high quality at all phases from recruitment to the last follow-up. All testing timepoints will be completed by the same assessor to ensure assessment coherence, and also to enhance participant retention. The participants can have information about their condition at all timepoints which will also promote their commitment to the study. No data will be collected after discontinuance of the study, however, all previously collected data will be included in the analysis.

### Data management {19}

The General Data Protection Regulation (EU GDPR), the data protection law, the guidelines of the Finnish National Board on Research Integrity (TENK) and the European Federation of Academies of Sciences and Humanities (ALLEA) will be used to guide the research integrity in the implementation of the research. The data protection law as well as the ethical instructions of JYU and the Regional Medical Research Ethics Committee of Wellbeing Services County of Central Finland will be followed in conducting the research and handling subjects in a vulnerable position. In this study, data is not transferred outside the EU/EEA area. The storage of personal data is regulated by legislation and good scientific practice. Personal data is collected only for this study and stored separately from all other data. All data is stored on a secured data server, that has been created for this study by JYU digital services.

Named members of the research group, the professional assessors, and the research therapists will have access to the personal data to the extent that is essential for their duties. A separate data management plan has been prepared, according to which the details of data management are handled during the study.

### Confidentiality {27}

All personal data collected in this study will be carefully handled as stated in a separate data management plan. Confidentiality will be maintained by pseudonymisation of all personal data, that is possible to pseudonymise. Video data or other data that cannot be pseudonymised will be accessed only by the researchers whose duties require working with the data. All other identifiers will be removed after which all data is stored with participant codes, and the code key can be only accessed by the PI.

### Plans for collection, laboratory evaluation and storage of biological specimens for genetic or molecular analysis in this trial/future use {33}

Not applicable as no biological specimens were collected in this trial.

## Statistical methods

### Statistical methods for primary and secondary outcomes {20a}

All statistical tests will be two-tailed with a significance level of 5%, and no adjustments will be made for multiplicity. Primary and secondary outcome measures at baseline, post-intervention, and at the first and second follow-ups will be treated as continuous dependent variables.

For the IIMT group, which also serves as the waiting-list control, we will assess clinical improvement by comparing their progress during the intervention period with that observed during the waiting period prior to treatment. Specifically, we will perform paired samples t-tests for each outcome measure, comparing the change from baseline 2 (6 weeks; pre-intervention) to post-intervention (12 weeks) against the change from baseline 1 (0 weeks; no intervention) to baseline 2 (6 weeks). Prior to analysis, we will assess the normality of the difference scores to ensure the assumptions of the t-test are met. This comparison will help isolate the treatment effect from natural fluctuations over time.

Since the participants are randomised either to the waiting list plus plain IIMT group or to one of the other three arms (B, C, and D), comparisons between plain IIMT and the waiting list are based on within-subject analyses rather than fully randomised group contrasts. No direct comparisons between the waiting list group and arms B, C, and D will be performed.

To evaluate the effects of VAT and ML on these outcomes, repeated-measures linear mixed-effects models will be applied [50]. A key advantage of this design is its ability to retain participants with missing data, ensuring that all participants contribute to the analysis. Additionally, they can account for baseline group differences, to avoid overestimating the effect size of treatment. VAT and ML will be included as fixed effects, with a random intercept for each participant to account for the dependency of repeated measurements. To control for baseline differences across conditions, treatment terms will be omitted from the model, allowing the effects of VAT and ML to be estimated through their interactions with time. As an exploratory analysis, the models will be extended to include a VAT × ML interaction term to examine potential synergistic effects between the two interventions.

In addition to evaluating treatment effects post-intervention and at follow-up time points, we will calculate an overall treatment effect over time (denoted as *B*). This will represent the raw mean difference associated with the presence versus absence of each factor. *B* will be derived by summing the regression coefficients corresponding to each condition across all time points [28]. To estimate effect sizes for each outcome, the overall treatment effect over time will be divided by the baseline standard deviation of the respective measure across all participants.

For each participant, a dichotomous treatment response variable will be calculated, defined as a reduction of at least 50% in CORE-OM scores between the baseline and post-intervention assessments. Fisher’s exact test and odds ratios will be calculated separately for VAT and ML. For statistically significant effects, clinical significance will be assessed by calculating the risk difference and the number needed to treat (NNT).

### Interim analyses {21b}

Not applicable, no interim analysis included.

### Methods for additional analyses (e.g. subgroup analysis) {20b}

In addition to the primary efficacy analysis, an adjusted efficacy analysis and two sensitivity analyses will be conducted. Each repeated-measures linear mixed-effects model for continuous outcomes will be adjusted for the following prognostic covariates: age group (18–34, 35–49, 50–65), gender, medication use (antidepressants, anxiolytics, or hypnotics). Therapists will be added as random effects. Model fit will be assessed through the Akaike Information Criterion (AIC) and Bayesian Information Criterion (BIC). The covariates that optimise these criteria will be retained in the final model. Two sensitivity analyses will be performed for the primary outcome: (1) a single imputation method using Last Observation Carried Forward (LOCF), which assumes no change for missing data, and (2) an analysis based on treatment as received.

### Methods in analysis to handle protocol non-adherence and any statistical methods to handle missing data {20c}

An intention-to-treat (ITT) approach will be employed, incorporating all available data regardless of whether the treatment was received as intended. Participants who discontinue the study before completing the intervention will be classified as dropouts.

For dichotomous variables (e.g., early study termination, treatment response), missing data will be imputed using a conservative approach in which participants who leave the study early will be assumed to have experienced a negative outcome (i.e., dropout or no response).

### Plans to give access to the full protocol, participant level-data and statistical code {31c}

For further research, the metadata description will be made available via an Open-Source database JYX (Jyväskylä University Digital Repository). If necessary, temporary access to data will be implemented through signed data transfer and co-operation agreements in accordance with the PI.

## Oversight and monitoring

### Composition of the coordinating centre and trial steering committee {5d}

The study is conducted and coordinated by the iMTDep research group that runs the trial and provides day to day support for the interventionists. This group is in weekly contact with the larger MMBB research group, which provides support and oversight. The trial steering committee meets once a year to discuss the progress of the study. The steering committee consists of three experts, who are from the fields of psychiatry, psychotherapy and neurology. There are also technical staff to provide support in technical issues. The IT services and the data administration and protection services and infrastructure of the University of Jyväskylä are also there to support the team when needed Fig. 1.

### Composition of the data monitoring committee, its role and reporting structure {21a}

We have not established a Data Monitoring Committee (DMC). We are, however, committed to monitoring the data throughout the study to ensure the scientific and ethical standards. There are four research team members (researchers and technical staff) responsible for data monitoring and three members from the organisation (specialists in data management and data protection) for additional support.

### Adverse event reporting and harms {22}

There are some identified unintended consequences or adverse events that can occur during the study.

1. Temporary weakening of the participants’ condition
  In therapy context with people with mental disorders there is always a risk for temporary weakening of the participants’ condition. There may be some illness specific fluctuation, but it is also possible that the intervention engages the participant in an emotional process that is experienced as unpleasant and disturbing. Levels of depression, anxiety and stress might also be temporarily increased. Going through difficult emotions and memories during assessment or intervention or disclosure of personal issues in front of cameras in therapy setting can cause psychological distress. Specific attention is thus paid to the clinical qualification, expertise, and training of the interventionists to minimise the risks in advance. Frequent clinical supervision is also offered throughout the study.
2. Issues dealing with Resonance Frequency Breathing (RFB) included in IIMT
  When asked to slow down their breathing, some people tend to breathe more deeply, which can lead to hyperventilation. We will therefore specifically instruct the participants to breath naturally and lightly and even more shallowly if they notice any symptoms of hyperventilation, such as light-headedness. In the previous RCT with RFB intervention [28] there were not any serious issues with RFB technique.
3. Issues dealing with vibroacoustic treatment
  Vibroacoustic treatment is generally considered safe and well tolerated medium for supporting relaxation and working with both physical and mental realm of sensations. The sensitivity of receiving vibroacoustic stimulation can be different between individuals. Possible overstimulation (felt as slight discomfort or nausea) can be usually avoided by using only mild levels of stimulation.
4. Technical issues
  The complexity of the study design may lead to technical issues, which in turn could cause inconvenience or even harm to participants. For example, they may have to wait longer than expected or fail to receive all the treatments intended for them.

All adverse events and harms will be collected and recorded in the participant’s clinical record in the main coordination file. PI will assess each adverse event and take appropriate actions if needed. Adverse events will be reported in the main publication of the study.

### Frequency and plans for auditing trial conduct {23}

A monthly report is provided for the management of the research centre. The steering committee is informed about the progress of the study yearly or in case additional attention is needed. The medical ethical board is informed about the main study results.

### Plans for communicating important protocol amendments to relevant parties (e.g. trial participants, ethical committee) {25}

Modifications concerning the research protocol and procedures as well as changes in staff members are communicated to the medical ethical board. Protocol amendments will be reported to ISRCTN Clinical Study Registry.

### Dissemination plans {31a}

Study results will be published in peer-reviewed journals and disseminated in conferences and other scientific meetings, and to the press.

## Discussion

The challenges of balancing work and personal life are increasingly perceived as pressuring, often leading to various mental health issues. Prolonged, untreated occupational stress can escalate into more severe conditions such as burnout, depression, and anxiety. Our ongoing research project represents our third RCT aimed at working-age adults with depression and anxiety. This time, we've decided to expand our focus to include work-related stress and exhaustion due to their significant comorbidity with depression and anxiety.

We have observed that a relatively short-term music therapy intervention (consisting of 12 to 20 sessions) significantly improves the condition of our participants [23, 28]. We hypothesise that this approach can also effectively address work-related stress and exhaustion due to their close links with depression and anxiety. Given the increased need for innovative treatments in this domain, music therapy stands out as a promising and effective option, providing possibilities for non-verbal expression and interaction.

Furthermore, there is a growing need to investigate the underlying mechanisms of music therapy. Consequently, we have incorporated two additional music therapy methods – VAT and receptive music therapy – into our model alongside improvisational music therapy, which remains our primary method. While verbal processing remains an option in our model, we have observed that non-verbal musical expression and interaction can complement and enhance verbal processing, facilitating emotional expression and regulation.

Our previous RCTs have demonstrated low dropout rates and relatively high treatment adherence. The current study differs in both its expanded inclusion criteria and the adoption of two new music therapy methods. We aim to maintain sample homogeneity and ensure that these changes do not dilute the overall efficacy of music therapy across different subject groups. We are eager to determine whether modifications to the original Integrative Improvisational Music Therapy (IIMT) model significantly enhance the overall effectiveness of music therapy. This aspect is crucial, as the field offers numerous music therapy methods, yet evidence regarding their relative efficacy remains limited.

## Trial status

The study was registered in ISRCTN Clinical Study Registry on 12.04.2024 (ISRCTN26812986). Recruitment started 05.06.2024 and is estimated to be complete by the end of December 2028. The current protocol is version 2 (date of approval: 20/04/2026).

## Supplementary Information


Supplementary Material 1.

## Data Availability

The PI will have access to the final dataset of the trial. The data will be stored according to the data management plan. The Regional Medical Ethics Committee will re-evaluate the need for continuing the data storage every five years.

## Abbreviations

iMTDep: Integrative Music Therapy for Depression-related Disorders
ISRCTN: The UK’s Clinical Study Registry
IIMT: Integrative Improvisational Music Therapy
DSM: Diagnostic and Statistical Manual of Mental Disorders
ICD-10: International Classification of Diseases, 10th Revision
JYU: University of Jyväskylä
RCT: Randomised Controlled Trial
ML: Music Listening
VAT: Vibroacoustic Treatment
TAU: Treatment as usual
RFB: Resonance Frequency Breathing
MADRS: Montgomery-Åsberg Depression Rating Scale
HADS-A: Anxiety subscale of the Hospital Anxiety and Depression Scale (HADS)
KEDS: Karolinska Exhaustion Disorder Scale
AUDIT: Alcohol Use Disorders Identification Test
HRV: Heart Rate Variability
CORE-OM: Clinical Outcomes in Routine Evaluation – Outcome Measure
HADS: Hospital Anxiety Depression Scale
BAT: Burnout Assessment Tool
BAT-12: Burnout Assessment Tool, 12-item version
RAND-36: 36-Item health survey
TAS-20: Toronto Alexithymia Scale
VAS: Visual Analogue Scale
EPS: Emotional Processing Scale
SEQ: Session Evaluation Questionnaire
PI: Principal Investigator
EU GDPR: European Union, General Data Protection Regulation
TENK: Finnish National Board on Research Integrity
ALLEA: European Federation of Academies of Sciences and Humanities
EU: European Union
EEA: European Economic Area
NNT: Number Needed to Treat
AIC: Akaike Information Criterion
BIC: Bayesian Information Criterion
LOCF: Last Observation Carried Forward
ITT: Intention to Treat
DMC: Data Monitoring Committee
MMBB: The Centre of Excellence in Music, Mind, Body and Brain
