# Supplementary material for: Integrative Music Therapy for Depression-related Disorders (iMTDep) – study protocol for a randomised controlled trial
Source: Trials. 2026 Jun 19;27:554. doi: 10.1186/s13063-026-09864-4 (PMC13527921; doi:10.1186/s13063-026-09864-4)
Supplement: Supplementary file 1 [file 13063_2026_9864_MOESM1_ESM.docx]

A template

University of Jyväskylä

*This is a template for the ethical informed consent to be requested from the participant of the study*

*As an ethical requirement, consent must always be obtained from the participant and it is recommended that it be requested in accordance with this template, in terms of content.*

INSTRUCTION: Use this template when you have chosen this option as the legal basis for the processing of personal data in the privacy notice: “Scientific research serving a public interest (GDPR, Article 6.1e, special personal data categories 9.2j)”

Responding to an online questionnaire: the participant must first have easy access to the information notice, the privacy notice and the text of the consent form. It is recommended that access to the questionnaire should not be given until the subject has taken some action to indicate that he/she has read the documents and understands that responding to the questionnaire is equivalent to giving consent. They confirm this, for example by clicking on a box.

Oral consent: the researcher must document consent in one way or another, for example by videotaping or recording when data are collected using these methods, or by documenting how and when consent is obtained.

Delete the instructions marked in grey (including these) once you have completed the template.

**CONSENT TO PARTICIPATE IN SCIENTIFIC RESEARCH**

*[Title of the study]*

I understand that participation in the study is voluntary and that I can pause or stop taking part in the study at any time without giving a reason, and there will be no negative consequences for me. The research materials collected about me up to the point of pausing or stopping may still be used in the study.

By giving my consent I accept that data and materials will be collected from me as described in the information sheet.

**[If necessary, add more details on what the subject consents to:]**

By giving my consent to participate in this study:

[*If applicable/remove when not applicable*]:

I consent to *[select: photographs/videos* being taken of me for research purposes, [select: but they will be processed in such a way that I cannot be identified / they will not be used in research results or publications.

Yes ☐	No ☐

[*If applicable/remove when not applicable*]:

I consent to my [*select:* name/identifiable photographs / identifiable videos of me being published in connection with publications and research data [**NOTE: only applicable** when there is a scientific reason to present the research data in an identifiable way, such as when interviewing a visual artist about his or her work, researching e.g. a celebrity athlete or a politician].

Yes ☐	No ☐

[*If applicable/remove when not applicable*]:

I consent to [*describe what is being asked to consent*]

Yes ☐	No ☐

[*If applicable/remove when not applicable*]:

I confirm that I will not participate in face-to-face data collection if I have flu symptoms, fever, am recovering from illness, or am feeling otherwise unwell.

Yes ☐

**I confirm** that I have received the information sheet about the the content of the study, how it will be conducted and what it means in my part, as well as the privacy notice. I have also had the opportunity to ask the researchers further questions. I have therefore been adequately informed about the content of the study, how it will be conducted and what it means for me, as well as about the processing of my personal data which are processed on the basis of public interest as set out in the law.

I have had sufficient time to consider my participation in the study. I have not been pressured or tempted to take part in the research.

**Yes ☐**

*[Choose an applicable option]:*

[*Consent form on paper*] Signature of the participant, name in block capitals, and date

[*Electronic questionnaire box*] By ticking this box, I agree to participate in this study / those sections where I have ticked "yes".

[*Oral consent: the researcher must document consent in one way or another, for example by videotaping or recording when data are collected using these methods, or by documenting how and when consent is obtained.]*

Contact details:

[*Contact details of the contact person*]

Paper or recorded consent is stored in accordance with data security guidelines, as is other personal data.

[Template updated 2/2024]

A concent form model of the imtdep-study

University of Jyväskylä

**CONSENT TO PARTICIPATE IN SCIENTIFIC RESEARCH**

Music therapy for working-age people with depression-related disorders – a randomised controlled trial combined with mixed methods research (iMTDep)

I have been asked to participate in the aforementioned scientific research that investigates the possibilities and mechanisms of music therapy in treatment of depression-related disorders.

**I confirm** that I have received the information sheet about the content of the study, how it will be conducted and what it means in my part, as well as the privacy notice. I have also had the opportunity to ask the researchers further questions. I have therefore been adequately informed about the content of the study, how it will be conducted and what it means for me, as well as about the processing of my personal data which are processed on the basis of public interest as set out in the law.

I understand that participation in the study is voluntary and that I can pause or stop taking part in the study at any time without giving a reason, and there will be no negative consequences for me. The research materials collected about me up to the point of pausing or stopping may still be used in the study.

I have had sufficient time to consider my participation in the study. I have received information about my rights, the purpose of the research and its conductment, and the pros and cons of the research. I have not been pressured or tempted to take part in the research.

By giving my consent, I accept that data and materials will be collected from me as described in the information sheet. After the research, the data is archived as described in the privacy notice. I know that my information is handled confidentially.

**I have considered the points above and I have decided** that I want to participate in the research.

**Yes ☐**

Additionally, I give my permission that:

I can be invited to an interview

Yes ☐	No ☐

after this research ends, I can be invited to a follow-up study related to the same topic

Yes ☐	No ☐

____________________________________________	_____________________

Name of the participant	Date of birth

_____________________________________________________________________________________

Address

______________________________	__________________________________________________

Phone number	Email

_____________________________	__________________________________________________

Date	Signature

Consent collected:

____________________________________________	______________________________

Name of the person responsible of the research	Date

_____________________________________________________

Signature of the person collecting the consent

Original signed consent and a copy of the information sheet will stay in the archive of the person responsible of the research. Paper or recorded consent is stored in accordance with data security guidelines, as is other personal data. Information sheet and a copy of the signed consent is given to the participant.

**Contact details:**

Coordinator

Markku Pöyhönen

The Department of Music, Art, and Culture Studies

[markku.i.poyhonen@jyu.fi](mailto:markku.i.poyhonen@jyu.fi)

040 570 7007

Doctoral researcher

Tiia-Liina Raittila

The Department of Music, Art, and Culture Studies

[tiia-liina.raittila@jyu.fi](mailto:tiia-liina.raittila@jyu.fi)

050 596 8722

Doctoral researcher

Marianne Taipale

The Department of Music, Art, and Culture Studies

[marianne.h.taipale@jyu.fi](mailto:marianne.h.taipale@jyu.fi)

050 428 4234

Associate professor

Esa Ala-Ruona

The Department of Music, Art, and Culture Studies

[esa.ala-ruona@jyu.fi](mailto:esa.ala-ruona@jyu.fi)

040 805 4297
